# What You Don’t Know About the Codex Can Hurt You: How Trade Policy Trumps Global Health Governance in Infant and Young Child Nutrition

**DOI:** 10.34172/ijhpm.2021.109

**Published:** 2021-08-24

**Authors:** Katheryn Russ, Phillip Baker, Michaela Byrd, Manho Kang, Rizki Nauli Siregar, Hammad Zahid, David McCoy

**Affiliations:** ^1^Economics Department, University of California, Davis, Davis, CA, USA.; ^2^Institute for Physical Activity and Nutrition, Deakin University, Geelong, VIC, Australia.; ^3^University of California, Davis, Davis, CA, USA.; ^4^Centre for Primary Care and Public Health, Queen Mary University, London, UK.

**Keywords:** Infant Formula, Commercial Milk Formulas, Trade Policy, Commercial Determinants of Health, Political Economy

## Abstract

**Background:** International food standards set by the Codex Alimentarius Commission (CAC), have become more prominent in international trade politics, since being referenced by various World Trade Organization (WTO) agreements. We examine how this impacts implementation of the World Health Organization (WHO) *International Code of Marketing of Breast-milk Substitutes*.

**Methods:** Using trade in commercial milk formulas (CMFs) as a case study, we collected detailed data on interventions across various WTO bodies between 1995 and 2019. We used language from these interventions to guide data collection on member state and observer positions during the CAC review of the *Codex Standard for Follow-up Formula* (CSFUF), and during CAC discussions on the relevance of WHO policies and guidelines.

**Results:** Exporting member states made 245 interventions regarding CMFs at the WTO, many citing deviations from standards set by the CAC. These did not occur in formal disputes, but in WTO Committee and Accession processes, toward many countries. In Thailand, complaints are linked to weakened regulation. Exporters also sought to narrow the *CSFUF* at the CAC in a way that is at odds with recommendations in the *International Code*. Tensions are growing more broadly within the CAC regarding relevance of WHO recommendations. Countries coordinated during WTO committee processes to advocate for reapportioning core WHO funding to the CAC and in order to further influence standard-setting.

**Conclusion:** The commercial interests of the baby food industry are magnifying inconsistencies between health guidelines set by the WHO, standard-setting at the CAC, and functions of the WTO. This poses serious concerns for countries’ abilities to regulate in the interests of public health, in this case to protect breastfeeding and its benefits for the health of infants, children and mothers.

## Background

The World Health Organization (WHO) recommends that “infants should be exclusively breastfed for the first six months of life to achieve optimal growth, development and health […and thereafter] receive nutritionally adequate and safe complementary foods while breastfeeding continues for up to two years of age or beyond.”^1^Few interventions can surpass breastfeeding as a positive health measure for children. Not breastfeeding is estimated to cause 595379 child (6 to 59 months) deaths annually from diarrhoea and pneumonia and 98243 adult deaths from breast and ovarian cancers and type-2 diabetes.^2,3^ Although the rate of exclusive breastfeeding in the first 6 months improved globally from 33% in 1995 to 42% in 2018,^4^ this is an insufficient rate of progress to meet the World Health Assembly’s (WHA’s) global target of 50% by 2025.

One factor contributing to low worldwide breastfeeding rates is the inappropriate marketing and aggressive promotion of breastmilk substitutes (also known as BMS), defined as foods marketed or otherwise represented as partial or total replacements for breastmilk, including any milk products marketed for consumption by children up to 36 months of age.^5^ Commercial milk formulas (CMFs) are the main BMS products marketed and consumed worldwide, including infant, specialised, follow-up and toddler categories. Numerous studies document the prevalence of inappropriate CMF marketing in all countries, irrespective of their development status.^6,7^ Exposure to such marketing—including product labelling practices, engagement with health professionals, and direct-to-consumer advertising—is associated with reduced breastfeeding initiation, exclusivity and duration, with women in many countries being more likely to recall CMF advertisements than information about the benefits of breastfeeding.^7-9^ Partially in consequence, global CMF sales have grown rapidly in recent decades, doubling from US$22.9 billion in 2005 to US$55.6 billion in 2019.^10-12^

The WHO-UNICEF (the United Nations Children’s Fund) *Global Strategy for Infant and Young Child Feeding*^1^ calls on governments and other actors to protect, promote, and support breastfeeding, including enacting and enforcing provisions of the 1981 * International Code of Marketing of Breast-milk Substitutes* and subsequent WHA resolutions, hereafter called the *International Code*. The * International Code *is a response to a longstanding concern that the aggressive marketing and promotion of CMF undermines breastfeeding and harms infant, child and maternal health in all countries.^13^ The *International Code* is further supported by state obligations under the *Convention on the Rights of the Child*,^14^ including Article 24 on the right to health and nutritious food. However, nearly one third of WHO member states have yet to adopt any provisions of the *International Code* into domestic law and less than one fifth have adopted all provisions.^15^In countries where provisions have been adopted, enforcement mechanisms are often insufficient to deter violations.^16^

In this paper, we provide new data documenting how countries adopting provisions of the *International Code* are increasingly challenged at the World Trade Organization (WTO) by exporters of dairy and CMF products. Exporting countries cite international standards set by a United Nations food safety advisory body called the Codex Alimentarius Commission (CAC) to challenge domestic regulation, while simultaneously engaging in crafting the standards used as grounds for their own complaints. Our analysis of overlapping WTO and CAC processes show how corporate interests at play in trade policy and the WTO system push for food standards that would limit countries’ ability to implement WHO guidelines for infant and young child feeding.

The treatment of restrictions on marketing of CMF by the WTO has not been systematically documented in the literature. We quantify the many ways that CMF- and dairy-exporting countries raise CMF-related complaints at the WTO, and how the complaints also influence domestic regulation of CMF marketing even in the absence of a formal case being filed at the national or international level. We then examine the CAC standard-setting process for follow-up formula (FUF), one type of CMF product, and describe how this process influences trade negotiations and disputes occurring under the auspices of the WTO. We conclude by emphasizing that the outcome of the (ongoing) revisions of the FUF standard will be key to whether or not countries will be able to regulate CMF marketing in accordance with the *International Code* and best practices for public health. In essence this paper examines the intersection between international trade governance and global health governance and shows how adoption of the *International Code* became subject to trade complaints.

###  The Intersection Between International Trade Governance and Global Health Governance

Thow et al call the intersection between global health and international trade governance a “regime complex.”^17^ The term regime complex refers to “…a set of overlapping and perhaps even contradictory regimes that share a common focus,” such that “…decisions made in one forum can be influenced, revised, or undermined by decisions and politics within a parallel or overlapping domestic or international forum (p. 330-331).”^18^ We demonstrate the relevance of the theory by examining how the separate regimes for global public health and trade policy interact with each other around the promotion of CMF trade, including the evolution, application, and enforcement of CMF standards.

**Figure 1:**
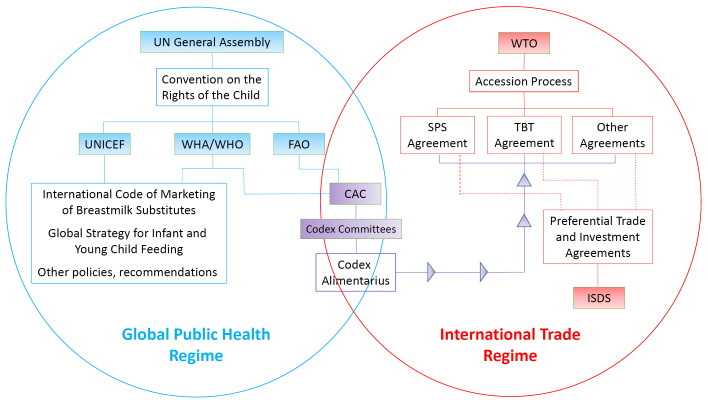
Stylistic Representation of the Regime Complex for Infant and Young Child Nutrition. Abbreviations: UN, United Nations; UNICEF, UN Children’s Fund; WHA, World Health Assembly; WHO, World Health Organization; FAO, Food and Agriculture Organization; CAC, Codex Alimentarius Commission; WTO, World Trade Organization; SPS, sanitary and phytosanitary; TBT, technical barriers to trade; ISDS, Investor-State Dispute Settlement.

Figure 1 is a stylized depiction of the regime complex, highlighting those elements that are central to infant feeding and foods. These organizations are split across two systems of actors and institutions, called regimes: the global public health regime and the international trade regime.

###  The Global Public Health Regime for CMF

The blue circle on the left in Figure 1 represents the global public health regime and the components of the regime that are central in promoting and protecting infant nutrition and child health. These include multilateral organizations like the United Nations, WHO, and UNICEF, as well as their policy instruments (the *Convention on the Rights of the Child*, the *International Code*, and the *Global Strategy for Infant and Young Child Feeding*). The WHO is governed by its member states, primarily through the WHA as its main governing body. Although the WHA has law making powers, it tends to govern global health through encouraging states to adopt international standards, norms and guidelines. The International Health Regulations and the Framework on Tobacco Control are two exceptions, cases of legally binding agreements defining countries’ rights and obligations to address infectious disease outbreaks and tobacco-related harms. Even in these cases, member countries have the option to reject the conventions by declining to ratify them.^19,20^

Although the WHA has the power to make international regulations on an opt-out basis, under pressure from industry and the US, it instead adopted the * International Code* in 1981 as a set of non-binding recommendations for WHO member countries to voluntarily adopt into national law.^20^ Every year, delegates to the WHA gather in plenary to review reports and vote on resolutions that have been drafted by various committees that collectively cover WHO’s mandate “to promote health and ease the burden of disease worldwide.” In doing so, the WHA has the authority to strengthen or weaken the *International Code* through additional resolutions, in response to new scientific evidence, and expert opinion gathered and generated by WHO staff and others. However recent resolutions intended to strengthen the * International Code* have been heavily contested.^21-24^

In 2016, the WHA also approved a Framework of Engagement with Non-State Actors (FENSA), allowing greater non-state actor participation, including by businesses and business-interest non-governmental organizations (NGOs)^[1]^. Stakeholders involved in advocating for breastfeeding under the *International Code* oppose loosening restrictions on participation of the CMF industry in WHA processes^25^ in light of the industry’s past history of working to weaken the Code, arguing that FENSA does not include adequate safeguards against conflicts of interest.^20,26,27^ At the same time, WHO’s approach to conflict-of-interest screening does not allow the relatively unfettered and direct participation by industry groups in health policy-making processes that is standard under the CAC’s committee system and have been protested by industry groups claiming it is extremely restrictive.^28,29^

###  The International Trade Regime for CMF

The red circle on the right in Figure 1 describes the international trade regime and the components of the regime that are central to the supply, distribution and marketing of infant food products. These include the WTO as an organization, with an Accession process involving initial negotiations with countries seeking to join, provisions of WTO Agreements that form the legal basis of complaints, and preferential trade and international investment agreements between two or more countries which reference or include provisions of the WTO Agreements. Preferential agreements may include special legal protections for cross-border investment called investor-state dispute settlement (ISDS) that may enable multinational companies to sue if the government adopts domestic regulations that adversely affect expected profits of affiliates on the ground.

Table 1 lists the particular bodies within the WTO relevant to CMF that operate within the red circle in Figure 1. Complaints about adoption of the *International Code* into domestic law can occur within a variety of WTO committees, including the Technical Barriers to Trade (TBT) Committee and Sanitary and Phytosanitary (SPS) Committee. Due to uncertainty surrounding how *Codex* standards might be interpreted or enforced in trade dispute settlement processes, national governments may also be subject to ‘regulatory chill,’ where just the possibility of trade sanctions or costly arbitration is enough to deter regulatory action,^31,32^ especially in preferential trade agreements containing investor protections in the form of ISDS provisions.

**Table 1:** WTO Bodies With CMF-Related Interventions 1995-2019

**WTO Committee, Council, or Body**	**Description**
Council on Trade in Goods	Oversees implementation of agreements related to goods trade, with 14 subsidiary bodies (13 Committees and a Working Party) covering various agreements and rules.
Committee on Agriculture	Under the Council for Trade in Goods, oversees the implementation of the WTO Agriculture Agreement, members can ask questions and express concerns about each other’s agricultural policies.
Committee on TBT	Under the Council for Trade in Goods, oversees the implementation of the WTO TBT Agreement, members can ask questions and express concerns about each other’s regulatory measures and standards that may restrict goods trade or discriminate against imports.
Committee on SPS issues	Under the Council for Trade in Goods, oversees the implementation of the WTO SPS Agreement, members can ask questions and express concerns about each other’s regulatory measures and standards related to food safety that may restrict trade or discriminate against imports.
Council for TRIPS	Oversees the implementation of the WTO TRIPS Agreement, members can ask questions and express concerns about each other’s policies related to the protection of intellectual property.
Trade Policy Review Body	A meeting of the General Council under special rules as part of the surveillance of WTO member countries’ trade policies and macroeconomic situation to increase policy transparency among members. The review centers around a report written by economists in the WTO Secretariat’s Office, called the Trade Policy Review.
Working Groups on Accession	Oversees negotiations with countries seeking to become a WTO member, a process known as Accession.

Abbreviations: WTO, World Trade Organization; CMF, commercial milk formula; SPS, sanitary and phytosanitary; TBT, technical barriers to trade; TRIPS, Trade-Related Aspects of Intellectual Property Rights. Source: WTO online archives, WTO Annual Report 2021.^30^

###  Where the Health and Trade Regimes Meet

Intersecting the two circles is the CAC and the *Codex Alimentarius*. A key function of the CAC is to harmonise standards across countries, “protecting the health of consumers and ensuring fair practices in the food trade (p. 4).”^33^ The CAC meets once a year; any member or associate member of the Food and Agriculture Organization of the United Nations (UN FAO) or WHO can participate.

The CAC is funded and administered by the UN FAO and the WHO, both parts of the UN System and the global public health regime. The body of international food standards that the CAC maintains, a text called the *Codex Alimentarius* (hereafter, *Codex*), contains benchmarks for all modern trade agreements. Both the WTO agreements and many preferential (bilateral and other regional) trade and investment agreements integrating provisions of these WTO agreements either implicitly or explicitly reference the *Codex*.^34,35^ This is why the *Codex* underlies the WTO Agreements in Figure 1. Harmonization of standards can help eliminate unnecessary hindrances to trade, preventing discrimination against imports and leading to efficiency gains.^36^ This is also why the *Codex*, the CAC Committees that craft its provisions, and the CAC itself as their governing body create an area of overlap between the global health regime and the trade policy regime. Explicit reference to the *Codex* in the WTO SPS agreement, combined with WTO jurisprudence that treats the *Codex* as one of the sets of standards implicitly referenced in the WTO TBT agreement, made the CAC the main arbiter of science for food standards, including manufacture and marketing of CMF, in trade policy.^37,38^ It is also referenced in WHA resolutions related to the *International* Code. For example, the 2016 WHA resolution 69.9 calls on the CAC to “give full consideration to WHO guidelines and recommendations, including the [ *International Code*] and relevant WHA resolutions.”^[2]^

**Figure 2:**
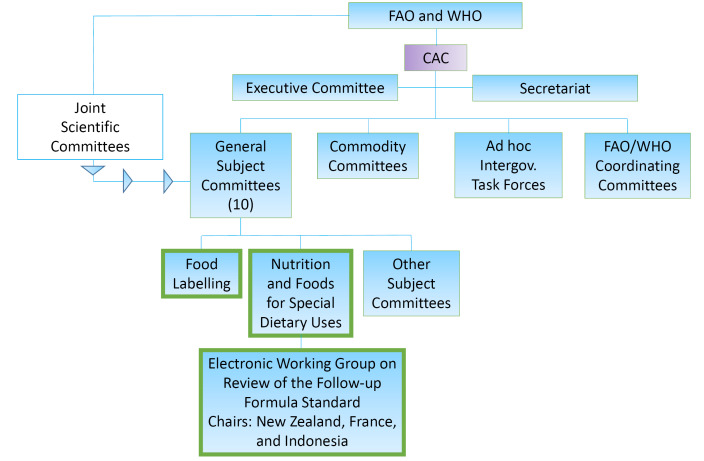
Organizational Structure of the CAC With Emphasis on CMF. Notes: Subject Committees with primary jurisdiction over CMF standards are the CCNFSDU and the CCFL. Abbreviations: WHO, World Health Organization; FAO, Food and Agriculture Organization; CAC, Codex Alimentarius Commission; CMF, Commercial milk formula.

Figure 2 is a stylized depiction of the CAC’s organizational structure with emphasis on bodies most relevant to CMF standard-setting. While the CAC itself has final decision-making authority on standards, its activities are overseen by an Executive Committee. Under the CAC’s jurisdiction are ten General Subject Committees, four Commodity Committees, six regional Coordinating Committees, and intermittent *ad hoc* Task Forces. Between sessions, the CAC’s Executive Committee makes decisions and it also makes proposals to the CAC for consideration in strategic planning and programming. The *Codex* standards themselves are crafted by the Subject Committees, covering issues such as food additives, contaminants, food labelling, food import and export inspection and certification and, pesticide residues, among others, and four Commodity Committees. This work is supported by two Joint Technical Committees to offer specific scientific advice and research, intermittent ad hoc task forces. The Coordinating Committees work to elevate priorities that may vary across geographic regions.

 Two Subject Committees have primary jurisdiction over CMF: the Codex Committee on Nutrition and Foods for Special Dietary Uses (CCNFSDU) and the Codex Committee on Food Labelling (CCFL). The CCNFSDU is charged with advising the CAC on nutrition issues and developing standards and guidelines for foods for special dietary uses. The CCFL is charged with drafting provisions related to food labelling, reviewing and amending provisions of other committees related to labelling, and studying misleading advertising practices for food. There also are Joint Scientific Committees administered by the FAO and WHO, which provide risk assessments and other technical advice to the CCNFSDU and other CAC Committees on issues like the safety of food additives (such as pectin or xanthan gum) and upper-bounds for added vitamins and minerals. The Coordinating Committees also recommend regional priorities that can influence standard-setting processes.

 Public and private non-state actors can participate as Observers during meetings of the CAC and its committees, as they craft food standards. Member states can vote during these processes, while Observers cannot. Yet Observers participate in meetings and post written comments, and can therefore be influential. Observers also can participate in specialized Working Groups which undertake some of the more detailed work of identifying appropriate data and language to shape the standards and which sometimes are charged with finding consensus on points of controversy. Industry groups therefore may participate directly in the process of crafting standards in CAC committees as Observers, as may consumer groups and public health advocates. As another channel of influence member state delegations also often contain industry representatives, in addition to government officials.

###  The CAC at the Center of Tensions Between Trade Policy and Public Health Governance 

The birth of the WTO in 1995 brought a stronger arbitration process for enforcement, coupled with strong language in the WTO Agreement on TBT specifying that domestic regulatory measures *not be more trade-restrictive than necessary to fulfil a legitimate objective*. It also required that assessing the appropriateness of any domestic regulatory measure be based on *risk*, using *available scientific and technical information*. This reached beyond the primary goal evident in earlier agreements to prevent domestic regulation from discriminating against foreign (imported) goods.^31,39,40^ The WTO SPS Agreement and references to international standards in the TBT Agreement have elevated the CAC as the principal judge of scientific evidence for trade in food, the arbiter weighing harmonization of standards to facilitate trade against health concerns where the two may conflict. Thow et al documented the enormous importance of the CAC’s *Codex* benchmarks in justifications of complaints at the WTO levied in regard to domestic policies regulating nutrition labelling in Chile, Ecuador, Indonesia, Peru and Thailand.^41^

As a recent assessment of these legal issues notes, the language in the WTO TBT Agreement on its face leaves room for domestic regulation in alignment with WHO’s guidelines and recommendations.^42^ Nevertheless, the shift that occurred with implementation of the WTO Agreements—reaching beyond primarily just preventing discrimination against imports, and providing a new Arbitration Body to facilitate stronger enforcement of international standards—has opened the door for countries to levy more complaints against domestic regulatory measures on the grounds of being “overly trade-restrictive” or “not science-based.”^31,43^ This introduces uncertainty for countries seeking to regulate but concerned about the costs of protracted litigation and the threat of punitive tariffs, whether or not sanctioned through arbitration.

###  Conflicts of Interest

The heightened legal weight of *Codex* standards under WTO rules since 1995 provide clear motivation for industry groups to exercise influence both as Observers and embedded within member state delegations in the CAC standard-setting process. The proportion of specific trade concerns related to food raised in WTO committees citing deviation from *Codex* standards increased more than five-fold between 2007 and 2016.^34^ It is now widely known that this presents “conflicts of interest.” The commercial interests of CMF manufacturers to maximize profits present a conflict of interest in any discussion of CMF regulation that may affect profits. *Codex* standards are meant to protect public health and facilitate fair practices in the food trade, but there are obvious incentives for industry participants (or member states lobbied by their domestic exporters and resident multinational firms) to work to structure standards that instead maximize profits in exporting industries.^44^ Thow et al explore in depth how commercial interests have influenced the deliberations for food labeling, making it likely that standards will conflict with WHO guidelines in a way that limits the ability of national governments to implement them without incurring the risk of inciting a trade dispute.^17^ Wieck and Grant present another example of how the competing interests of exporters of chocolate from countries with different levels of cadmium in their soils is shaping deliberations over the *Codex* standard for cadmium in chocolate products.^45^

In contrast, the WHO has tighter limits on industry participation in national delegations and as observers. While representatives from CMF producers can attend various CAC committee meetings as part of member state delegations, Chapter V, Article 11 of the WHO Constitution explicitly states that national delegates to its governing body, the WHA, should be a *health ministry official* with appropriate technical qualifications. A Credentialing Committee reviews the credentials of proposed delegates prior to WHA meetings.^46^

Given the differential screening for conflicts of interest, one could expect less restrictive policy guidelines on the marketing of CMF-related products in *Codex* standards than in the *International Code*. Koletzko and Shamir discuss the adoption and revision of standards for CMF under the purview of the CCNFSDU.^47^ Reporting on a meeting to update CMF standards in 2005, the authors describe the considerable influence of commercial enterprises in the deliberation of the committee, such that “scientific and medical arguments may be unduly influenced by commercial considerations (p. 621).” In this paper, we show how this tension plays out within WTO trade policy processes, connecting it directly to the path of current deliberations within CAC Committees over draft revisions for the FUF standard, and an example of the impact on Thailand’s domestic regulation of CMF marketing.

## Key Messages

**Implications for policy makers**

- Public health advocates and governments wishing to safeguard space for national public health policy, particularly in infant and young child nutrition, should strengthen participation by health agencies and organizations in the Codex Alimentarius Commission (CAC), the United Nations food standard-setting body.
- World Trade Organization (WTO) Agreements and have made standards for food safety and marketing set by the CAC a stronger constraint on the scope for domestic public health regulation.
- The ability of national governments to implement World Health Organization (WHO) recommendations for protecting breastfeeding by restricting inappropriate marketing of commercial milk formulas (CMFs) is at risk of being weakened during the ongoing review of the *Codex Standard for Follow-up Formula *(CSFUF), in a way that would make challenges to national policy measures at the WTO harder to defeat.
- Industry participation is much less restricted within the CAC compared to the WHO, creating greater scope for private conflicts of interest to enter into international standard-setting and motivating exporters to argue against using WHO guidelines to inform standards for infant and young child nutrition in CAC processes.

**Implications for public**

The ability of national governments to implement public health policies aligned with World Health Organization (WHO) guidelines is at risk of being trumped by trade policy, including for infant and young child nutrition. World Trade Organization (WTO) agreements adopted globally in 1995 have centralized international standards set by the Codex Alimentarius Commission (CAC) as legal benchmarks. Industry interests are freer to participate in policy-making processes and have stronger influence at the CAC compared to the WHO, leading to standards sometimes at odds with WHO recommendations. Governments and public health advocates in civil society wishing to protect and promote breastfeeding by implementing the WHO’s *International Code of Marketing of Breast-milk Substitutes*—or to preserve the scope for other national health regulations, more generally—may wish to consider taking a more active role in CAC processes.

## Methods

 The analysis involved three steps: first, data collection from WTO documents; second, data collection from CAC documents; and third, data analysis.

###  Collecting Data on Positions of Actors Within the WTO

First, we examined the workings of institutions involved in shaping global trade policy as it affects CMF. To do this, we assembled a database of CMF-related interventions at the WTO through text searches of the WTO online archives, which include meeting summaries and other documents. We defined an intervention as a question, request, complaint, or expression of concern about restrictions or proposed restrictions on the marketing, distribution, or sales of CMF in a member or applicant country during a WTO committee meeting, council meeting, or process. We then identified the set of interventions related to infant formula or CMFs as those with explicit mention of infant formula or CMF. Since the focus of our study is trade policy, we conducted an especially exhaustive search for interventions 1995 to 2019 that were made publicly available by September 2020. The nature of our text search and the way we transcribed text into coded interventions is described in detail in Supplementary file 1.

###  Collecting Data on Positions of Actors Within the CAC Committees With Jurisdiction Over CMF 

Since the data on WTO interventions heavily cited the *Codex* and international standards, we then searched records documenting recent revisions of CMF-related standards at the CAC relevant to complaints we observed in WTO proceedings. We categorized and quantified various types of recent interventions pertaining to CMF sales and marketing in the main committees with jurisdiction over CMF (the CCNFSDU and the CCFL). Based on the focus of complaints at the WTO seeking to make a distinction between regulation of infant formula versus FUF, we chose to focus on a recent review of the *Codex Standard for Follow-up Formula* (CSFUF) as a key point of intersection between WTO and CAC processes. We therefore assembled data on negotiating positions related to implementation of the *International Code *within the FUF standard review.

Unfortunately, documentation on such interventions is limited to country comments and very general meeting summaries. Full deliberations are not transparent to the degree that one sees in WTO documents. Much deliberation likely also happens in-person during meeting breaks in hallways and over meals. The deliberations of the Electronic Working Group tasked with leading the *CSFUF* review are completely obscured. So our analysis without a doubt captures only a portion of the full scale of the arguments taking place.

###  Data Analysis

 We generated descriptive statistics to illustrate the trade and public health regime complex on breastfeeding and CMF consumption by tallying the types and frequency of interventions at the WTO and creating charts with data detailing major themes or alliances in deliberative processes at the CAC and the WTO. A replication package with data and Stata v13 code used to export aggregates into spreadsheets for charts is available from the corresponding author upon request.

## Results

 We organized the findings as follows. First, we examined the treatment of CMF within the trade policy regime, presenting the new data on trade-related interventions involving CMF both inside and outside the WTO. Second, we used those data to examine a deliberative process involving the ongoing revision of the CSFUF that played out in parallel with complaints by exporter countries at the WTO.

###  The WTO and the Regulation of CMF Marketing

 Concerns related to the marketing, labelling and safety of CMF have been expressed in a variety of ways by WTO members. In this section, we use newly collected data from the WTO archives to document the nature of CMF-related interventions during WTO processes.

We discovered CMF-related interventions in all of the WTO arenas described in Table 1. Interventions occurred not only between WTO members, but in the WTO Working Groups on Accession, meaning that WTO members asked detailed questions about domestic regulation of CMF products even as countries applied for WTO membership. Table 2 shows that the majority of CMF-related interventions toward WTO members (post-Accession) has taken place in the TBT Committee and during Trade Policy Reviews, but at least as many interventions were made toward non-member countries during the Accession process.

**Table 2:** Interventions Regarding CMF Made by WTO Members, 1995-2019

**Committee, Council, or Process**	**Interventions**
Agriculture	15
Council for Trade in Goods	1
SPS	6
TBT	58
Trade Policy Review	29
TRIPS Council	1
Accession Process	135
**Total**	**245**

Abbreviations: WTO, World Trade Organization; CMF, commercial milk formula; SPS, sanitary and phytosanitary; TBT, technical barriers to trade; TRIPS, Trade-Related Aspects of Intellectual Property Rights. Notes: “Intervention” defined as a complaint or expression of concern about restrictions or proposed restrictions on the marketing, distribution, or sales of CMF in one member country registered by a committee or council, or by a delegation from another country, either in person or in writing.

###  Pre-Accession

Not all pre-Accession proceedings were made publicly available. However, Table 2 shows 135 instances of countries receiving questions during “Question and Response” processes and initial negotiating rights (INRs) made public in WTO website archives. INRs involve negotiations by individual members of the WTO with a country being considered for Accession over access to its domestic market for a specific product^[3]^. 30 of 36 countries who participated as an applicant in the Accession process between 1996 and 2019 experienced one or both of these types of interventions, with 18 of these 30 countries asked specific questions about their domestic treatment of CMF products. More than half of the 18 received multiple questions, with Saudi Arabia, Ukraine, and the Russian Federation asked to answer between 15 and 20 CMF-related questions. Ten countries applying for Accession engaged in INRs with a WTO member for one or more CMF products. Most of these INRs were granted by Accession applicants who were low- or middle-income countries to high-income WTO member states (the United States, Australia, and the European Union) requesting additional access for their CMF exports.

###  Post-Accession

**Figure 3:**
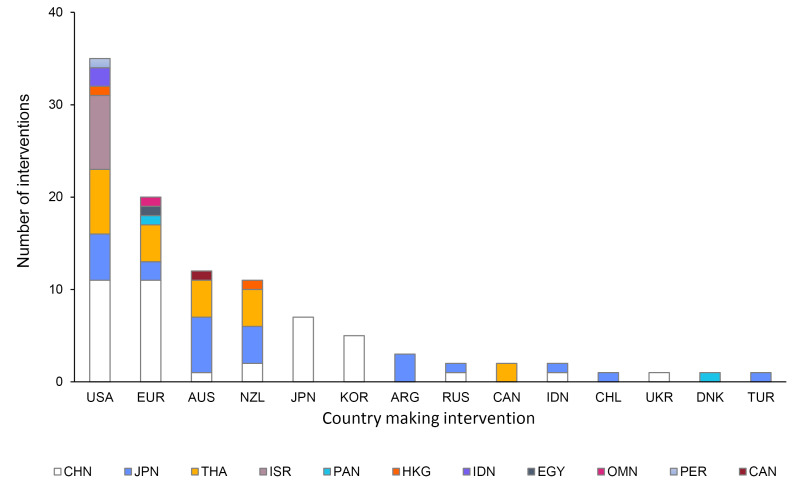
Member Countries Making vs Receiving WTO Interventions Involving CMF, 1996-2019. Notes: WTO members along horizontal axis intervene against members denoted by colors. Abbreviations: WTO, World Trade Organization; CMF, commercial milk formula.

In total, 110 interventions related to CMF have taken place across a number of bodies within the WTO since 1995. Figure 3 shows countries making CMF-related interventions toward WTO members (post-accession only). Members of the WTO making the interventions are labelled along the horizontal axis, with the WTO members on the receiving end of the interventions denoted by colors. The chart shows that the vast majority of concerns are voiced by high-income countries who are major exporters of CMF and dry milk powder (the United States, European Union, Australia, and New Zealand), voicing concerns about policies implemented or proposed in countries with lower levels of income per capita.

###  Quantifying Use of New TBT Criteria for Domestic Public Health Regulation

**Figure 4:**
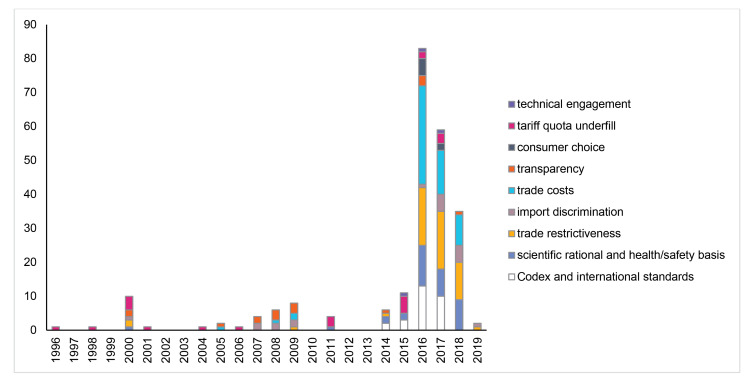
Percentage of CMF-Related Interventions Mentioning Particular Concerns, 1996-2019. Notes: Data collected from WTO online archives. Percentages do not add up to 100 percent, as interventions may mention more than one concern and this list is not exhaustive. Abbreviation: CMF, commercial milk formula.

Figure 4 shows that the number of interventions has grown significantly in the last 5 years. In addition, their content has changed. Prior to 2014, concerns about measures affecting CMF centred on questions about the transparency of measures and whether they were discriminatory toward imports. Since then, interventions have been dominated by TBT-related concerns such as compliance with Codex/international standards, whether there is a scientific basis for regulation, and whether regulation is trade-restrictive or involves costs of compliance for foreign suppliers.

###  The Regime Complex and Health Policy in a CMF Importer – the Case of Thailand

**Figure 5:**
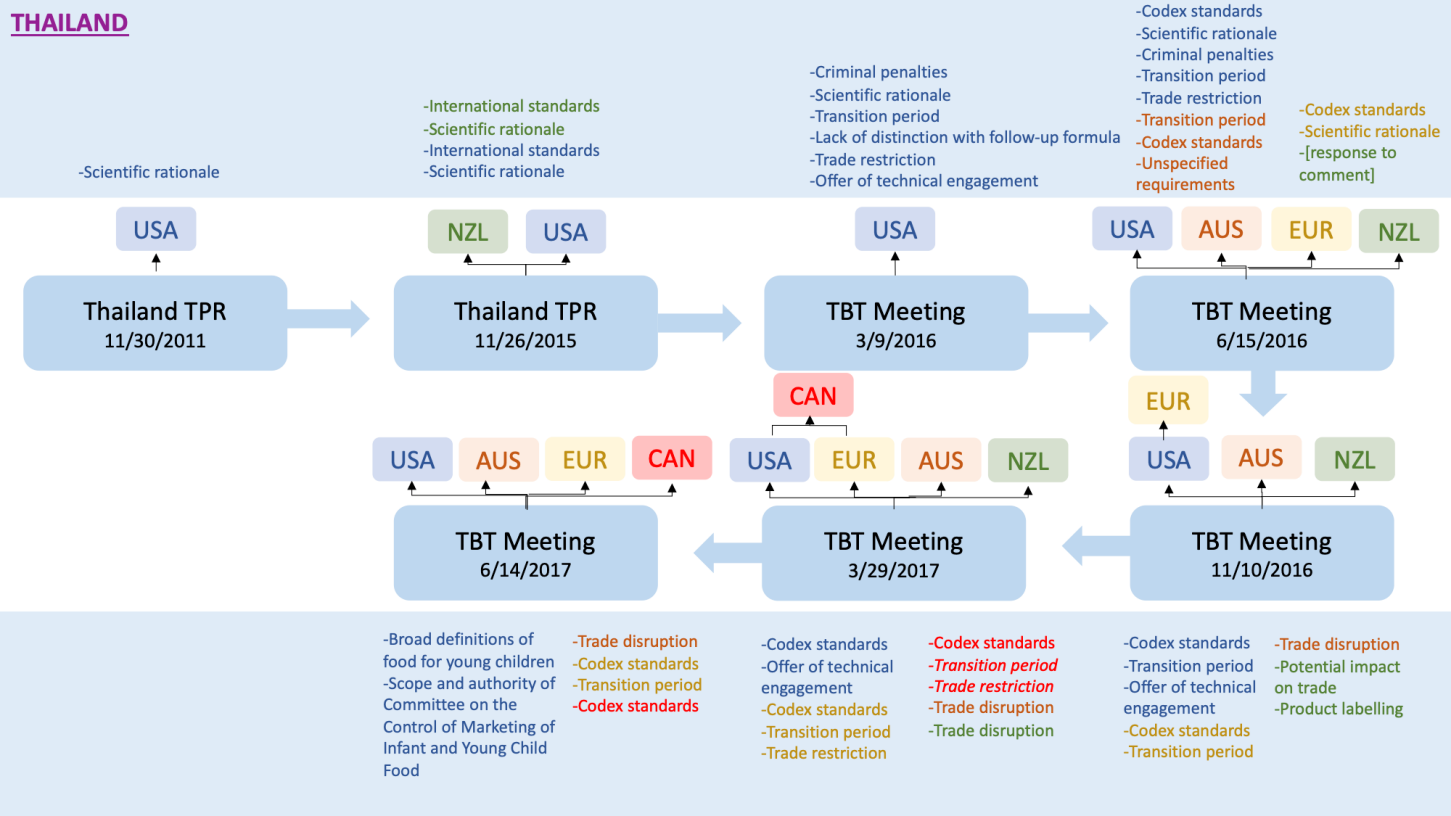
Interventions at the WTO involving Thailand’s Domestic Regulation of CMF. Notes: Data from WTO online archives. Text in light blue bars at top and bottom are descriptors of the complaints levied by countries shown as having intervened against Thailand’s proposed legislation in each meeting. Interventions are denoted by an arrow from a meeting to an intervening country. Text is matched to intervening country by color. Abbreviations: WTO, World Trade Organization; CMF, commercial milk formula; TBT, technical barriers to trade.

Thailand’s “Milk Code” illustrates in detail how interventions within WTO processes occur as a country designs domestic legislation to implement the *International Code*. Figure 5 is a flowchart showing the progression of interventions against Thailand after it introduced domestic legislation to restrict inappropriate marketing of CMF. The first interventions about the Milk Code consisted of concerns expressed about the proposed legislation during the WTO Trade Policy Review of Thailand in 2015^[4]^. Both the United States and New Zealand suggested that the proposed legislation did not conform to international standards and requested an explanation of its scientific rationale.

Criticisms of Thailand’s proposed legislation continued in the TBT Committee. US delegates expanded their concerns at the March 2016 meeting, posing questions about the scientific rationale, the use of criminal penalties, and the application of restrictions to FUF. At the June meeting, Australia, the European Union, and New Zealand formed a coalition with the United States to echo these concerns, with Canada joining later. The coalition explicitly and repeatedly referenced *Codex* standards in its concerns about whether there was adequate scientific rationale for the proposed legislation. In November 2016, the coalition added concerns about Thailand’s requirements for product labelling, with the United States asking whether measures complied with Thailand’s own laws on food labelling. At the March 2017 TBT Committee meeting, three of the five coalition members again voiced concerns about compliance with *Codex* standards and four complained about trade-restrictiveness. Almost every one of the 21 interventions over the 20-month processes involved a questioning of Thailand’s scientific rationale or a complaint about the proposed legislation not conforming to *Codex* standards.

This is a rare case where we can see the original proposed legislation and the final version of the legislation as passed as reported by the US Department of Agriculture Foreign Agricultural Service. Both the US Department of Agriculture and the US Trade Representative^48^ detailed the changes integrated into to the proposed law before it was passed that may benefit US exporters. In particular, the US Department of Agriculture Foreign Agricultural Service^49^ noted that:

*“The approved legislation limits the prohibition on advertising to just food for infants and supplementary food for infants. Advertising for food for young children is now permitted as long it does not cause the public to believe the product is for infants or cause the public to believe that the product was suitable for feeding infants. The approved legislation reduces the maximum criminal penalty of imprisonment from 3 years to 1 year and the maximum fine from 300000 baht to 100000 baht for violations of the advertising prohibitions.*”^[5]^

 These changes weakened the law by exempting drinks for children 13 months and older from being regulated in the same way as infant formula, including allowing demonstrations of FUF in health centers, and by reducing criminal penalties for violations of marketing restrictions. This exemption can be a loophole for the marketing technique of ‘cross-promotion.’

###  Standard-Setting in CAC Committees

Our data show that CMF-related interventions at the WTO have emphasized scientific rationale, international standards, and the health or safety basis for measures, for all of which the *Codex* standards are benchmarks. Thus, our data on WTO interventions show that apart from the *International Code*, the *Codex* effectively serves as the second major plank of the global public health regime for infant and young child nutrition.

**Figure 6:**
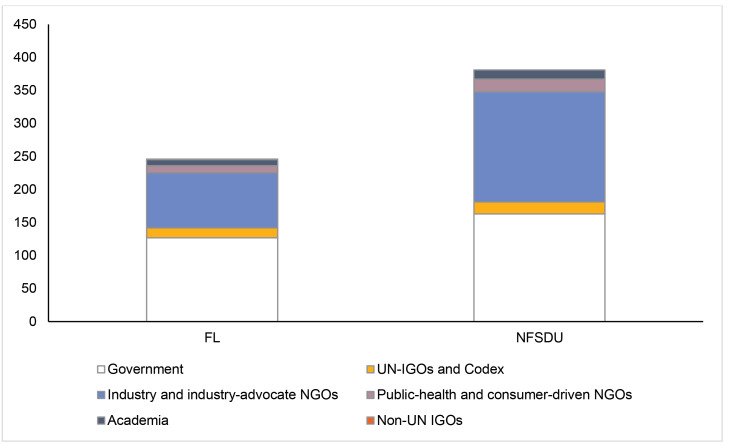
Attendance in Codex Committees Reviewing FUF Standard in 2019. Notes: Data gathered from participant lists in CAC REP19/FL and REP20/NFSDU. Notes: Data gathered from participant lists in CAC REP19/FL and REP20/NFSDU. Abbreviations: NGO, non-governmental Organization; IGO, intergovernmental organization; UN, United Nations; FUF, follow-up formula.

Figure 6 shows the prominence of industry participation during the annual meetings of these two committees in 2019. Industry representatives within member country government delegations (with full voting rights) as well as industry-advocate observers made up more than 40% of total participants in the CCNFSDU meeting—nearly matching the number of officials from national governments, WHO, FAO, and the CAC Secretariat combined. Industry participants made up exactly one third of all attendees in the CCFL meeting. Often, industry participants attend as part of a member government delegation.

###  Tensions Between Trade and Health During CMF Standard-Setting at the CAC

The process for establishing standards for CMF products through the Codex committees is partially observable in meeting summaries and written comments posted by participants. Table 3 presents some of the issues under contention during an ongoing review of the *CSFUF *(Codex Stan 156-1987), which began in 2017. Two of three co-chairs were representatives of top CMF producer-exporter countries (New Zealand and France), a common practice in CAC committee processes.^44^

**Table 3:** Debates During the Review of the CSFF (CXS 156-1987) in the CCNFSDU

**Issue**	**Perspectives**	**Position**	**Proponents at Meetings of CCNFSDU**
Labelling of products of young children, aged 12-36 months	Public health	Do not allow "formula" or "formulated" in proposed product name, "[formulated] drink for young children."	Brazil, Cambodia, Colombia, Cote d'Ivoire, EU, HKI, Jamaica, Nepal, Sri Lanka
	Industry-preferred	Include "formulated" or "formula" in the product name, "[formulated] drink for young children."	Australia, Indonesia, India, ISDI, Malaysia, New Zealand, USA
Definition of products for young children, aged 12-36mos (Section B, 2.1.1)	Public health	Products for young children are manufactured for use as BMS (defined in WHA 69.9).	Cambodia, Cote d'Ivoire, Ecuador, HKI, IBFAN, India, Jamaica, Lao PDR, Mali, Mexico, Nepal, Philippines, Senegal, UNICEF, WPHNA
	Industry-preferred	Products for young children are not manufactured for use as BMS.	Argentina, Australia, Brazil, Canada, Colombia, Costa Rica, EU, Ghana, Indonesia, ISDI, Malaysia, New Zealand, Switzerland, Thailand, USA
How to structure the standard for Follow-On Formula, which can be for labelled for ages 6-36 months	Public health	Products for older infants (6-12 months) and products for young children (12-36 months) are governed by one standard with two parts.	Cambodia, Ecuador, EU, HKI, IBFAN, Nepal, Sri Lanka, UNICEF
	Industry-preferred	Products for older infants (6-12 months) and products for young children (12-36 months) are governed by two separate standards.	Argentina, Australia, Brazil, Canada, Colombia, ISDI, Malaysia, New Zealand, Switzerland, USA
Preamble for standard	Public health	Reference the International Code and subsequent resolutions (like WHA 69.9) in the Preamble.	Cambodia, Ecuador, Egypt, Jamaica, Nepal, Philippines, HKI, IBFAN, UNICEF
	Industry-preferred	Do not reference sources "external to Codex" in the Preamble.	USA
Sweeteners	Public health	Additives with sweet taste should not be used.	EU, HKI, India, Lao PDR, Kenya, S Korea, Mali, Mexico, Morocco, Nigeria, Panama, WPHNA
	Industry-preferred	Additives with sweet taste should not explicitly be restricted in the standard.	Chile, Costa Rica, ISDI, Russia
Cross-promotion (Section B, 9.6.4)	Public health	Cross-promotion must be prohibited.	Burkina Faso, Cambodia, Chile, Ecuador, HKI, IBFAN, India, Kenya, Kuwait, Lao PDR, Mali, Mexico, Nepal, Nigeria, Senegal, Sri Lanka, Switzerland, Uruguay, UNICEF, WPHNA
	Industry-preferred	Cross-promotion is not clearly defined hence should not be regulated by the standard.	Argentina, Australia, Brazil, Costa Rica, Dominican Republic, EU, Guatemala, IDF/FIL, Indonesia, ISDI, Korea, New Zealand, Panama, Peru, USA, Vietnam
	Mixed	Amend the provision on cross-promotion.	Australia, Canada, Colombia, Costa Rica, Indonesia, Malaysia, New Zealand

Abbreviations: CCNFSDU, Codex Committee on Nutrition and Foods for Special Dietary Uses; CSFUF, Codex Standard for Follow-up Formula; BMS, breastmilk substitutes; WHA, World Health Assembly; EU, European Union; UNICEF, United Nations Children’s Fund; HKI, Hellen Keller International; ISDI, International Special Dietary Foods Industries; IBFAN, International Baby Foods Action Network; IDF/FIL, International Dairy Federation; WPHNA, World Public Health Nutrition Association. Notes: Positions from CS/NFSDU 18/40/4, CS/NFSDU 18/40/5, CS/NFSDU 19/41/5, NFSDU/41, and REP20/NFSDU (including all appendices and comments).

 During the meetings, members were divided on whether the uniform standard for products made for persons 6 to 36 months of age should be divided into two standards: one for formula for “older infants” (6-12 months) and one for formula for “young children” (12-36 months). As a compromise, the CCNFSDU decided to have one standard with two sections. This allows setting higher nutritional standards for “formula for older infants.” Before the change, formula for infants 6-12 months was held to the weaker uniform standards for all formula products in the 6-36 months range. In the revised draft, products for older infants have higher nutritional standards than products for toddlers. Participants also debated other aspects of composition, such as whether ingredients with a sweet taste, certain types of sugars, or caps on sugar content should be allowed.

Members currently have not agreed on what to call products meant for young children 12 to 36 months old. Industry actors and governments in countries where the CMF industry is influential have advocated for the words “formula” or “formulated” to be included. Public health proponents have argued that this would confuse consumers, risking inappropriate use for babies 0-12 months old and falsely suggesting nutritional adequacy or special health benefits.^[6]^ The debate over the name of these products is also closely related to a debate over *cross-promotion*. Cross-promotion refers to the marketing of follow-up and toddler formula that simultaneously promotes use of infant formula by labelling and branding the products in a similar way. This issue was addressed in 2016, through resolution WHA 69.9 and supporting WHO technical guidance on ending the inappropriate promotion of foods for infants and young children.

The relevance of the *International Code* to the *CSFUF* has also been a point of contention. Proponents of breastfeeding have sought to reference the *International Code* in the Preamble to the revised Codex standard and frequently cite the *International Code* and related supporting evidence to justify their positions. As seen in Table 3, CMF industry proponents argued not to cite the *International Code* in the *CSFUF*. They also rejected a number of provisions of the *International Code* during deliberation of the standard, including the prohibition of cross-promotion of infant formula and products for young children and the categorisation and regulation of products for young children (13-36 months) as BMS. Public health advocates succeeded in ensuring that a footnote was inserted into the standard noting that “In some countries these products are regulated as breast milk substitutes,”^50^ most likely to protect the right to regulate FUF as recommended by the *International Code* (under WHA Resolution 69.9) if the standard is used as a benchmark in trade disputes. The US opposed the footnote, arguing that the use of footnotes when it is difficult to reach consensus is generally problematic, as Codex standards should be “global in nature.” A similar footnote allowing the limit on sugar content in the standards for FUF allows “national and/or regional authorities” to limit non-lactose carbohydrates to 1.25 g/100 kcal (less than the *Codex* maximum of 2.5 g/100 kcal) currently also remains contested.^50^

###  Tensions Over Standards and Governance Between the WHO, Codex, and WTO

Looking more broadly across CAC and WTO records further illustrates overlap between the two regimes. At the 2016 annual meeting of the Codex Executive Committee, representatives of the FAO and WHO voiced concern that CAC members are not weighing FAO and WHO policies in their work.^51^ FAO and WHO officials submitted a background document citing eight examples of Codex guidance or processes—including on drinking-water quality, nutrition labelling, and CMF—that were in conflict with FAO/WHO policies or UN public health initiatives.^52^ The meeting ended without discussion of the issue, but the topic was raised again at the next meeting in 2017. The WHO representative pointed out that the effects of this inconsistency were that WTO member states who are trying to implement measures adopted by the WHA are being accused in the WTO TBT Committee of creating trade barriers by failing to comply with the *Codex*.^53^ A WHO representative argued that international food standard setting was also a function delegated to WHO in its Constitution. Thus, the representative argued that even though *Codex* standards are cited in the WTO SPS Agreement, WHO standards could be considered as international standards referred to in the WTO TBT Agreement.^53,54^

###  Coordination Within the WTO to Influence CAC Governance

CAC member countries disagreeing with the WHO and FAO took up the discussion during a WTO SPS Committee meeting in November 2017. The US argued in support of CAC independence from WHO and FAO as key to ensuring fair trade in food products.^55^ Argentina and the US argued again at a WTO SPS Committee meeting in March 2018 that because the *Codex* is explicitly cited in the WTO SPS Agreement, it takes precedence over FAO and WHO standards, using this as an argument for *Codex* independence from its parent bodies, with member-driven processes and decisions.^56^

Some WTO members concurrently organized *within* WTO committee deliberations to influence CAC governance, funding, and standard-setting procedures. The FAO and WHO provide USD$12 million in technical support for Codex processes largely through their Scientific Advice Program, but within the CAC, some members have expressed concern that these funds are not from sustainable sources and are too little, requiring the CAC to turn often to member states and even industry for donations.^53^ (WHO officials have described hesitation developing within the WHO to increase funding for CAC processes because Codex guidelines “were not always developed consistent with WHO policies, guidelines, and recommendations”^57^). During an SPS Committee meeting, Canada, the EU, and the US called for members to urge the WHO and FAO to set aside sustainable funding for scientific advice, possibly by diverting funds from the WHO core budget. They also encouraged members to contribute financially to support CAC risk-assessment bodies.^56^

###  Coordination Within the WTO to Influence FUF Standard-Setting at the CAC

A communication from Mexico to the WTO TRIPS (Trade-Related Aspects of Intellectual Property Rights) Council in November 2019 highlights another example of other components of the trade regime influencing processes or standard-setting by CAC. Several weeks before the annual 2019 CCNFSDU meeting, Mexico alerted the WTO membership to proposals being considered in the CAC committees aimed at prohibiting cross-promotion of BMS and FUF under the same brand. Mexico argued that the proposals, which were submitted based on scientific evidence of potential harm to infants and consistent with WHA resolutions, should be screened for consistency with the protection of the value of trademarks under TRIPS and that TRIPS should take precedence in the design of the *Codex* standard.^58^

Table 3 documents how countries eventually lined up on this issue at the CCNFSDU. In the end, while the Committee agreed that labelling of FUF would not be allowed to “refer” to infant formula, it did not explicitly prohibit the labelling of infant and follow-on formula from resembling each other. The CCNFSDU also decided to remove all reference to the term “cross-promotion” from the draft standard.^54^ Industry is still striving to have the word “formula” or “formulated” included in the name for drinks for young children, which public health advocates argue supports cross-promotion.

## Discussion

Many low- and middle-income economies are striving to restrict inappropriate marketing of CMF by implementing the WHO’s *International Code*. But in doing so, they run the risk of opposition from CMF exporters that is channelled through the institutions of the international trade regime, which may include costly legal battles and ultimately retaliatory tariffs. At the center of this turmoil between the international public health and trade regimes is the CAC, which sets standards for the composition, labelling, and marketing of CMF products and therefore is ideally situated to help resolve these tensions by providing international benchmarks.

In principle, the CAC is administered by the FAO and WHO, both of which have strong procedures to limit industry influence or screen for conflicts of interest, with a mandate to protect public health. In practice, CAC processes and standards lack strong screening for conflicts of interest.^44^ Conflicts of interest among industry participants need not always outweigh the benefits of added information from industry input into health policy.^59^ However, our analysis of the review of the *CSFUF* shows that CMF and milk-powder exporting countries and industry actors flooding the CCNFSDU as observers and as member-country delegates strive to shape standards in conflict with WHO policies and guidelines in ways that can harm breastfeeding, and thus harm infant, child and maternal health. The success of industry interests in shaping parts of *Codex* standards in ways that conflict with WHO recommendations strongly suggests that industry has more influence at the CAC than in other parts of the public health regime shown in Figure 1—namely, the UN bodies that have committed to support the *International Code*—or in countries with political will to adopt the *International Code* in domestic regulation.

In the current review of the standard for FUF, the right to regulate marketing of CMF products for young children 13-36 months as BMS in the same way as products for infants 0 to 12 months, as stipulated in the *International Code* (under WHA Resolution 69.9), hangs on a contested footnote acknowledging that “some countries” do so. In principle, this acknowledgment in the *Codex* that some countries regulate products for young children as CMF—if it survives final deliberations in the FUF review and is not deleted in future reviews—provides grounds to argue that such regulations are consistent with international standards. It is also crucial to prevent cross-marketing. Research shows that even in advanced economies, cross-marketing can lead parents to feed infants with follow-up and toddler formula instead of the infant formula, which has stricter nutritional requirements. The confusion of parents puts infant health at risk.^60-62^

Yet this footnote that allows national regulation to prevent cross-marketing is still only in draft form. We see Thailand’s attempt to adopt similar restrictions in its Milk Code thwarted after a barrage of sub-arbitration level interventions within the WTO TBT Committee. Our example of the proposed versus final Milk Code in Thailand demonstrates that trade complaints referencing the *Codex* need not reach arbitration to be effective in watering down legislation. It further shows that explicit language formally acknowledging that countries may regulate FUF in the same way as formula for infants 0-12 months is essential in the *Codex* standard to preserve the feasibility of domestic implementation of all provisions of the *International Code*. Our analysis also shows that at the same time exporting countries at the WTO were contesting adoption of recommendations from the *International Code* into the Thailand’s proposed Milk Code to help prevent cross-marketing of toddler formulas with formula for infants, exporters and industry Observers were working to narrow the *Codex* standard for FUF in ways that would strengthen the legal grounds for these complaints under trade law, and continue to do so. The ability to restrict sugars to less than 2.5 g/100 kcal or prevent companies from adding sucrose to FUFs also hangs by a contested footnote.

Mexico’s communication to the TRIPS Council is an unusually clear example of how, in practice, the politics of trade influences the *Codex* standards, rather than only “available scientific and technical information” (specified within the WTO TBT Agreement) as the yardstick to determine the appropriateness of domestic regulatory measures for public health. WTO committees have become a forum for coordination of interventions within the CAC and to coordinate interventions in the administration of the CAC by the WHO to the point of diverting funds from the WHO’s core budget.

Additional challenges for advocates of the *International Code* lie in the mounting battles across multiple CAC committees over cross-promotion, food packaging, and cautionary nutrition labelling, standards administered by the CCFL. The CCNFSDU consulted heavily with the CCFL to ensure that proposed revisions to the standard for FUF would be consistent with standards on labelling. These consultations resulted in replacing some text with references to the labelling standards set by the CCFL that also can be reviewed and revised over time, subject to the same tensions seen in the review of the standard for FUF. In a parallel but separate process, the CCFL has been drafting guidelines for ‘Front of Package Labelling’ since 2018.^17,63-65^ Member countries have subsequently expressed concern that the evolving new Codex standard^65^ is already in conflict with WHO guiding principles, first drafted in 2017. For instance, WHO guiding principles expressly urge that CMF products contain no front-of-pack nutritional claims,^66^ but a provision exempting CMF products from the new *Codex* front-of-package labelling standard is still under contention within CCFL’s deliberations.^65^

Lester notes that the closely-related push in trade policy toward insisting that food safety regulations be “science-based” presents additional challenges. The move creates even more stringent constraints upon domestic regulation by challenging regulations that may not only exist to discriminate against exports, but also those that exist to protect animal welfare or the environment.^43^ Equally problematic, science evolves and scientists may have differing approaches to assessing health risks.

Since 2016, the Codex Executive Committee has been urgently pushing the WHO to provide increased funding for an “enhanced work programme” to harmonize risk assessment across the FAO, the WHO, and the CAC’s joint scientific committees, focusing on risk-based assessments for food safety standards. Part of the agenda is to push national governments to adopt the same risk-based approach to food safety. The issue of harmonization of standards and its institutional context within the regime complex for public health and trade deserves much more research to understand the costs and benefits of such initiatives. Maggi and Ossa show that under conditions where lobbies have sufficient influence over international standards relative to domestic standards, trade agreements requiring harmonized standards can be detrimental to economic welfare.^67^

 In the current setting, the arbiters of scientific processes are likely to become increasingly subject to conflicts of interest. For example, we observe above instances of WTO members in the SPS Committee and the TRIPS Council organizing to influence the governance, structure, and funding of the CAC and its scientific advice, rather than working exclusively through their country counterpart delegates to the parent bodies, the FAO and WHO, where restrictions on participation of industry interests are tighter. Organizing at WTO to marshal alternative sources of funding or encourage mandatory allocations from WHO core funding may become a way to further weaken CAC observance of FAO and WHO health guidelines and introduce trade policy aims as a bigger influence in scientific processes and deliberations. If the United States ever reduced or withdrew funding from WHO, as it recently threatened, and diverted a portion of these funds directly to the CAC, this in particular could weaken the commitment of the CAC to the public health priorities of its mandate.

 Industry influence within the WHO itself also is likely to intensify through new avenues for direct participation. Inflows of private-sector funding may become even more important if member countries like the US curtail contributions. There is no doubt that activities of both the CAC and the WHO are resource-constrained and that financing could be strengthened, including from the private sector. However, coordinating within an organization focused primarily on commercial interests (the WTO) to influence the balance of existing funds allocated to the CAC versus WHO both undermines the global health governance regime and puts commercial interests above public health.

There are ways to ensure that food standards fulfil their central objective of protecting consumer health. First, harmonization of standards through the CAC would be less subject to such conflicts of interests and more likely to be aligned with public health objectives if health ministries, rather than ministries of agriculture and commerce, were acknowledged leaders in national Codex offices and delegations. Government officials with technical competencies in health fields or from health ministries are best placed to lead national offices and national delegations to the CAC and its committees, similar to those that lead country delegations to WHO. Countries may wish to scrutinize the common practice of embedding industry stakeholders in national delegations. Second, greater representation of public health advocates among observer groups could ensure that of health objectives take priority in the standard-setting process. Currently, public health advocates are far outnumbered by industry representatives as Observers. Civil society groups can participate on equal footing with industry groups in CAC processes, but we see evidence in Figure 6 suggesting that industry resources and personnel to do so currently far exceed those from public health and consumer advocates. Baker et al describe the scale of financing behind private-sector groups working to influence standard-setting and trade policy.^68^

Third, CAC documentation of committee proceedings could be more detailed and transparent. All records that we draw from have the advantage of being publicly available, but it would be helpful for public understanding and future research if the CAC provided more transparent records of both the committee meetings and email or other written deliberations during the proceedings of Electronic Working Groups. Full formal affiliations of individuals in both members state delegations and among observers were often difficult to identify. Documentation of funding and work within the Joint Scientific Committee for Food Additives related to advice given to the CCNFSDU on safety of additives like xanthan gum and pectin likewise was almost non-existent, beyond a summary in the Joint Scientific Committee for Food Additives annual report on its activities. So our analysis of the full scope of the role of commercial interests in shaping the *CSFUF* is necessarily incomplete.

 Finally, while industry can play a role in optimal standard-setting through the provision of data and technical expertise, much clearer principles must be formed and enforced to discriminate between provision of data and expertise versus direct participation in steering the internal standard-setting processes themselves. Stronger and routinized screening for conflicts of interest in both national positions and in reviews of draft standards at each stage of processing could safeguard against failures or weaknesses in any of these efforts. Coordination of interventions in CAC standard-setting and WHO funding within WTO committees (where health experts may not be present and exporter-country business interests dominate) merits close scrutiny and evaluation for legal and policy coherence.

If instead the current situation persists, the treatment of *Codex* standards as a ceiling rather than a floor for public health policy measures under the WTO Agreements becomes likely to lead to unnecessary and increasing harm. We see this risk above in the realm of infant and young child nutrition, where it threatens to prevent countries from taking effective measures to prohibit cross-promotion and limit the sugar content of drinks for young children claiming to be specially formulated for them.

## Acknowledgements

 The authors thank Ethan Feilich for research assistance and Karin Lapping, Roger Mathisen, and Elizabeth Zehner for helpful comments.

## Ethical issues

 No human subjects were involved in this research.

## Competing interests

 KR reports a grant from Alive & Thrive/FHI 360 during the conduct of the study.

## Authors’ contributions

 KR devised the project, supervising and participating in all data collection, analysis, and interpretation. She drafted the article. PB contributed portions of the text and the unifying theme of the “regime complex.” PB and DM made substantial contributions to the design of portions of the work and revised the work critically for important intellectual content. MB, MK, RNS, and HZ collected data and made substantial contributions to analysis and interpretation of the data.

## Funding

 KR received research funding from Alive & Thrive/FHI-360.

## Authors’ affiliations

^1^Economics Department, University of California, Davis, Davis, CA, USA. ^2^Institute for Physical Activity and Nutrition, Deakin University, Geelong, VIC, Australia. ^3^University of California, Davis, Davis, CA, USA. ^4^Centre for Primary Care and Public Health, Queen Mary University, London, UK.

## Supplementary files

Supplementary file 1. Compiling Data on Interventions at the WTO.

## Endnotes


^[1]^Stakeholders involved in advocating for breastfeeding under the *International Code* opposed participation of the CMF industry in WHA processes because of the clear conflicts of interest it would involve^25^ and the industry’s past history of working to weaken the initial provisions of the Code, and then circulating a watered-down interpretation of the *International Code* after it was adopted.^20^

^[2]^ A companion report for that resolution^5^ cites relevant *Codex* provisions including the ‘commodity-specific’ 1981 *Standard on Infant Formula (CXS 72-1981)*, the 1987 *Standard on Follow-up Formula (CXS 156-1987)* and a number of ‘horizontal standards’ including the *Standard for Labelling of and Claims for Foods for Special Medical Purposes (CXS 180-1991)* under the CCFL.^69^

^[3]^ Table S.1.5 in Supplementary file 1 presents data by country receiving questions or requests for INRs.

^[4]^This suggests that Thailand did not notify the WTO of its proposed policy when it was drafted, an observation confirmed within a 2018 report to Congress by the US Trade Representative.^48^

^[5]^The web address where this document was publicly posted is no longer works, so we post it at: https://katherynruss.weebly.com/publications.html.

^[6]^Public health advocates note that the drinks often have excessive levels of sugar and are not necessary or especially beneficial to health compared to, for instance, less expensive cow’s milk.^70^

