# Supplementary material for: What You Don’t Know About the Codex Can Hurt You: How Trade Policy Trumps Global Health Governance in Infant and Young Child Nutrition
Source: Int J Health Policy Manag. 2021 Aug 24;10(12):983–97. doi: 10.34172/ijhpm.2021.109 (PMC9309967; doi:10.34172/ijhpm.2021.109)
Supplement: Supplementary file 1 — Compiling Data on Interventions at the WTO. [file ijhpm-10-983-s001.pdf]

Article title: What You Don't Know About the Codex Can Hurt You: How Trade Policy Trumps Global Health Governance in Infant and Young Child Nutrition

Journal name: International Journal of Health Policy and Management (IJHPM)

Authors' information: Katheryn Russ[1]*, Phillip Baker[2], Michaela Byrd[3], Manho Kang[3], Rizki Nauli Siregar[3], Hammad Zahid[3], David McCoy[4]

[1]Economics Department, University of California, Davis, Davis, CA, USA.

[2]Institute for Physical Activity and Nutrition, Deakin University, Geelong, VIC, Australia.

[3]University of California, Davis, Davis, CA, USA.

[4]Centre for Primary Care and Public Health, Queen Mary University, London, UK

(*corresponding author: knruss@ucdavis.edu)

Supplementary file 1. Compiling Data on Interventions at the WTO

S.1.1 Overview

We searched through all WTO official documents on docs.wto.org that are unrestricted and freely available to the general public (coded as "U" by the WTO) and those that have been derestricted and released to the public (coded as "D" by the WTO). We focused on questions raised by members, councils and working groups on all topics regarding infant formula or breast-milk substitutes. In this regard, we did not code replies or unilateral notifications by members.

S.1.2 Collection of data on interventions raised to members

We coded infant-formula related interventions by members for the period 1995 to 2019 that are publicly available by 4 September 2020. We defined an intervention as a question or request during a WTO committee or council process. We then identified the set of interventions related to infant formula or breastmilk substitutes as those with explicit mention of infant formula or breastmilk substitute. We used the following keywords to find interventions related to infant formula:

1. breastmilk NOT infant
2. baby formula NOT infant
3. baby food NOT infant
4. breast milk NOT infant

We discovered interventions raised in meetings of Technical Barriers to Trade (TBT) Committee, Sanitary and Phytosanitary Measures (SPS) Committee, Agriculture Committee,

Trade Policy Review (TPR) Body of the General Council, and Trade-related Aspects of Intellectual Property Rights (TRIPS) Council. At the WTO, the Ministerial Conference is the governing body for the General Council, under whom all of the other Councils, Committees, Bodies, Working Groups, and Working Parties operate. The Ministerial Conference meets roughly every two years and the General Council is authorized to act on its behalf, making day-to-day decisions between the meetings of the Ministerial Conference. Table S.1.2 describes the various arenas within the WTO where we find that BMS-related interventions take place.

Table S.1.2 Description of WTO Arenas with BMS-Related Interventions 1995-2019

**WTO Committee, Council, or Body**	**Description**
Council on Trade in Goods	Oversees implementation of agreements related to goods trade, with 14 subsidiary bodies (13 Committees and a Working Party) covering various agreements and rules.
Committee on Agriculture	Under the Council for Trade in Goods, oversees the implementation of the WTO Agriculture Agreement, members can ask questions and express concerns about each other's agricultural policies
Committee on Technical Barriers to Trade	Under the Council for Trade in Goods, oversees the implementation of the WTO Technical Barriers to Trade Agreement, members can ask questions and express concerns about each other's regulatory measures and standards that may restrict goods trade or discriminate against imports
Committee on Sanitary and Phytosanitary issues	Under the Council for Trade in Goods, oversees the implementation of the WTO Sanitary and Phytosanitary Agreement, members can ask questions and express concerns about each other's regulatory measures and standards related to food safety that may restrict trade or discriminate against imports
Council for Trade-Related Aspects of Intellectual Property Rights (TRIPS)	Oversees the implementation of the WTO TRIPS Agreement, members can ask questions and express concerns about each other's policies related to the protection of intellectual property
Trade Policy Review Body	A meeting of the General Council under special rules as part of the surveillance of WTO member countries' trade policies and macroeconomic situation to increase policy transparency among members. The review centers around a report written by economists in the WTO Secretariat's Office.
Working Groups on Accession	Oversees negotiations with countries seeking to become a WTO member, a process known as Accession

We coded the content of the complaint or question within each one of these interventions and identify the type of the interventions. Table S.1.2 defines variables on content of interventions. Each variable has values of 0 and 1. Each intervention is coded as 1 for a particular variable if the content match the definition of the variable, and 0 otherwise.

Table S.1.3 explains the variables on types of requests stated in the interventions. Each variable has values of 0 and 1. Each intervention is coded as 1 for a particular variable if the content match the definition of the variable, and 0 otherwise. An intervention can fit have values of 1 for one or more types of interventions.

Table S.1.3 Categorization of Complaint in BMS-Related Interventions at the WTO

**Variables on Content of Interventions**	**Description**
trans	lack of transparency/no notification
discrim	import discrimination
time	transition time
science	scientific rationale
health	health/safety basis
duplic	duplicate regulation
codex	harmony with Codex Alimentarius
standards	harmony with international standards
trade-restrictive	trade restriction, trade disruption and/or unnecessary/arbitrary restriction
choice	consumer choice
cost	costliness
tariff	tariff quota underfill
engage	offer of technical engagement

Table S.1.4 Type of Request in BMS-Related Interventions at the WTO

**Variables on Types of Requests**	**Description**
clarify	clarification/notification
mod	modification
recon	reconsideration
extend	transition time
other	other type of request or comment

S.1.3 Collection of data on interventions raised to acceding countries

In addition to the interventions between WTO members in the Committees and Councils above, for interventions during accession processes, we coded the provisions of Initial Negotiating Rights (INRs). The INRs, described below, are opportunities to negotiate tariff concessions on a particular product. Because they usually are granted by the acceding country upon the request of a WTO member, we consider the existence of an INR as evidence of an intervention. We used all unrestricted and derestricted documents from inactive accession processes, i.e. for WTO members who engaged accession processes with documents available (derestricted) by 4 September 2020. There are 36 acceded countries that become members of the WTO after its formation in 1995, shown in Table S.1.4.

We collected interventions by searching through accession documents using the following keywords, then manually inspecting each to assure direct relevance to BMS. Results were compiled into a spreadsheet and available in the replication package.

1. infant
2. formula
3. breast milk
4. breastmilk
5. baby

We collect two types of accession interventions. First, we include questions raised by countries or members of accession working groups as interventions. Second, we also collect interventions in the form of provisions of INRs, both in draft documents and official documents. Since INRs are negotiated bilaterally, we cannot document the negotiation process as well as question and replies behind the provisions of the INRs. Result were compiled in a spreadsheet and are available in the replication package.

S.1.4 Background on Initial Negotiating Rights (INRs)

There are three separate tracks of accession meetings in the WTO. They are:

1. Multilateral process
2. Plurilateral process
3. Bilateral process

In the multilateral process, the acceding country provides a Memorandum on the Foreign Trade Regime (MFTR) which describes its economic condition and trade policies. In this process, the question-and-replies process is recorded in a Factual Summary document. Unfortunately, not all

documents in this process were unrestricted by the end of this study's search period. Our results summarize data from all that we could access through the WTO online archives.

Table S.1.4 Countries Completing Accession to the WTO 1995-2019, Un-/Derestricted

Country	Date of Accession	Link to Accession Documents
Afghanistan	7/29/2016	https://www.wto.org/english/thewto_e/acc_e/a1_afghanistan_e.htm
Liberia	7/14/2016	https://www.wto.org/english/thewto_e/acc_e/a1_liberia_e.htm
Kazakhstan	11/30/2015	https://www.wto.org/english/thewto_e/acc_e/a1_kazakhstan_e.htm
Seychelles	4/26/2015	https://www.wto.org/english/thewto_e/acc_e/a1_seychelles_e.htm
Yemen	6/26/2014	https://www.wto.org/english/thewto_e/acc_e/a1_yemen_e.htm
Tajikistan	3/2/2013	https://www.wto.org/english/thewto_e/acc_e/a1_tajikistan_e.htm
Lao PDR	2/2/2013	https://www.wto.org/english/thewto_e/acc_e/a1_laos_e.htm
Vanuatu	8/24/2012	https://www.wto.org/english/thewto_e/acc_e/a1_vanuatu_e.htm
Russian Fed.	8/22/2012	https://www.wto.org/english/thewto_e/acc_e/a1_russie_e.htm
Samoa	5/10/2012	https://www.wto.org/english/thewto_e/acc_e/a1_samoa_e.htm
Montenegro	4/29/2012	https://www.wto.org/english/thewto_e/acc_e/a1_montenegro_e.htm
Cabo Verde	7/23/2008	https://www.wto.org/english/thewto_e/acc_e/a1_capvert_e.htm
Ukraine	5/16/2008	https://www.wto.org/english/thewto_e/acc_e/a1_ukraine_e.htm
Tonga	7/27/2007	https://www.wto.org/english/thewto_e/acc_e/a1_tonga_e.htm
Viet Nam	1/11/2007	https://www.wto.org/english/thewto_e/acc_e/a1_vietnam_e.htm
Saudi Arabia	12/11/2005	https://www.wto.org/english/thewto_e/acc_e/a1_arabie_saoudite_e.htm
Cambodia	10/13/2004	https://www.wto.org/english/thewto_e/acc_e/a1_cambodge_e.htm
Nepal	4/23/2004	https://www.wto.org/english/thewto_e/acc_e/a1_nepal_e.htm
N. Macedonia	4/4/2003	https://www.wto.org/english/thewto_e/acc_e/a1_macedoine_e.htm
Armenia	2/5/2003	https://www.wto.org/english/thewto_e/acc_e/a1_armenie_e.htm
Chinese Taipei	1/1/2002	https://www.wto.org/english/thewto_e/acc_e/a1_taipei_chinois_e.htm
China	12/11/2001	https://www.wto.org/english/thewto_e/acc_e/a1_chine_e.htm
Moldova	7/26/2001	https://www.wto.org/english/thewto_e/acc_e/a1_moldova_e.htm
Lithuania	5/31/2001	https://www.wto.org/english/thewto_e/acc_e/a1_lituanie_e.htm
Croatia	11/30/2000	https://www.wto.org/english/thewto_e/acc_e/a1_croatia_e.htm
Oman	11/9/2000	https://www.wto.org/english/thewto_e/acc_e/a1_oman_e.htm
Albania	9/8/2000	https://www.wto.org/english/thewto_e/acc_e/a1_albania_e.htm
Georgia	6/14/2000	https://www.wto.org/english/thewto_e/acc_e/a1_georgia_e.htm
Jordan	4/11/2000	https://www.wto.org/english/thewto_e/acc_e/a1_jordan_e.htm
Estonia	11/13/1999	https://www.wto.org/english/thewto_e/acc_e/a1_estonia_e.htm
Latvia	2/10/1999	https://www.wto.org/english/thewto_e/acc_e/a1_latvia_e.htm
Kyrgyz Rep.	12/20/1998	https://www.wto.org/english/thewto_e/acc_e/a1_kyrgyz_republic_e.htm
Panama	9/6/1997	https://www.wto.org/english/thewto_e/acc_e/a1_panama_e.htm

Country	Date of Accession	Link to Accession Documents
Mongolia	1/29/1997	https://www.wto.org/english/thewto_e/acc_e/a1_mongolia_e.htm
Bulgaria	12/1/1996	https://www.wto.org/english/thewto_e/acc_e/a1_bulgaria_e.htm
Ecuador	1/21/1996	https://www.wto.org/english/thewto_e/acc_e/a1_ecuador_e.htm

The acceding country provides an initial proposed tariff schedule for goods and services to start the bilateral process. The acceding country can, upon request, grant individual WTO members an Initial Negotiating Right (INR). This INR grants holders the opportunity to negotiate concessions on specific tariff lines, supplementary to the proposed tariff schedule in the multilateral process. For more details on INRs, see the General Agreement on Tariffs and Trade Article XXVIII (p. 938-941) and the WTO Accession Division Note 14-25/Rev.1.

Table S.1.5 presents data on the number of BMS-related questions asked of and number of INRs granted by countries applying to accede to the WTO.

Table S.1.5 Interventions by WTO Members during the Accession Process, 1995-2019

Country	Interventions		Total
	Questions	Products with INRs	
Afghanistan	0	2	2
Albania	0	2	2
Azerbaijan	1	0	1
Bulgaria	1	0	1
China	0	1	1
Chinese Taipei	0	1	1
Croatia	0	2	2
Ecuador	0	1	1
Estonia	1	2	3
Jordan	4	0	4
Kazakhstan	4	12	16
Kyrgyz Republic	0	2	2
Lao	1	0	1
Latvia	1	1	2
Liberia	0	2	2
Lithuania	0	2	2
Nepal	2	1	3
N. Macedonia	3	2	5
Oman	3	1	4
Russian Fed.	20	5	25
Samoa	2	0	2
Saudi Arabia	15	0	15
Seychelles	1	2	3
Tajikistan	2	0	2
Tonga	1	0	1
Ukraine	17	0	17
Uzbekistan	3	0	3
Vanuatu	0	1	1
Vietnam	0	5	5
**Total**	**82**	**53**	**135**

Notes: Data sourced from WTO online archives; interventions include those in draft documents.
